# Comparing Augmentative Plating and Exchange Nailing for the Treatment of Nonunion of Femoral Shaft Fracture after Intramedullary Nailing: A Meta‐analysis

**DOI:** 10.1111/os.12580

**Published:** 2020-01-01

**Authors:** Yao‐feng Jin, Hai‐chao Xu, Zhong‐hai Shen, Xue‐kang Pan, Hui Xie

**Affiliations:** ^1^ Department of Orthopaedics Surgery The Second Affiliated Hospital of Jiaxing University Zhejiang China

**Keywords:** Augmentative plating, Exchanging nailing, Femoral shaft fractures, Intramedullary nailing, Nonunion

## Abstract

**Objective:**

The aim of this meta‐analysis was to systematically evaluate the efficacy of augmentative plating (AP) and exchange nailing (EN) in the treatment of nonunion of femoral shaft fracture.

**Methods:**

For the present meta‐analysis, PubMed, EMBASE, and the Cochrane Library were searched to identify relevant articles up to April 2019. Two investigators independently evaluated the quality of original publications following the guidelines proposed by the Cochrane Handbook. Data were extracted from the studies and analyzed using Review Manager 5.3.

**Results:**

Five studies were included in this meta‐analysis, with a total of 506 patients. There were 232 patients in the AP group and 276 patients in the EN group. The AP group was associated with higher union rate (*OR*, 11.66; 95% *CI*, 4.31–31.50; *P* < 0.01), shorter union time (*SMD*, −1.10; 95% *CI*, −2.09 to −0.11; *P* = 0.03), shorter operation time (*SMD*, −0.55; 95% *CI*, −0.88 to −0.21; *P* < 0.01), less blood loss (*SMD*, −1.72; 95% *CI*, −3.33 to −0.11; *P* < 0.01), and fewer complications (*OR*, −0.11; 95% *CI*, −0.16 to −0.07; *P* < 0.01) than the EN group.

**Conclusion:**

The results of the meta‐analysis showed that AP is found to be superior for nonunion of femoral shaft fractures in both intraoperatively (ie, shorter operation time and less blood loss) and postoperatively (ie, higher union rate, shorter union time, and lower complication rate). Overall, AP was superior to EN in the treatment of nonunion of femoral shaft fractures after intramedullary nailing (IMN).

## Introduction

The femur is the strongest long tubular bone in the human body, as well as the main weight‐bearing bone of the lower extremities. It is prone to fracture when struck by strong external forces, such as in car accidents, and especially in falling injuries. Femoral shaft fractures are commonly caused by strong external forces, especially falling injuries^1^. With the progress of social modernization, the incidence of femoral shaft fractures has increased dramatically in about 5 years. Intramedullary nailing (IMN) has achieved good results in the treatment of adult femoral shaft fractures due advantages such as resulting in less trauma. However, poor blood supply and severe soft tissue damage often result from inappropriate surgical operations or deep infection, leading to delayed nonunion or bone nonunion. The incidence of nonunion of femoral fractures after trauma is 5%–10%^2^. However, some studies have found that the incidence of nonunion caused by IMN of the femoral shaft has even reached above 10%^3^. The occurrence of nonunion can cause obvious pain symptoms and seriously affect the daily life of patients.

The appropriate fixation methods for nonunion of femoral shaft fractures after IMN are still under discussion. The main methods include plate fixation, exchange nailing (EN), and bone grafting.^4^, ^5^, ^6^. EN is considered a reliable surgical technique for nonunion after IMN because of the high healing rate^7^. The long‐term effect of intramedullary nailing on nonunion has also been reported. In addition, the success rate of augmentative plating (AP) in the treatment of nonunion has been reported as 100%^8^.

In the treatment of nonunion of femoral shaft fractures, AP and EN have their respective advantages and disadvantages. Several studies^9^, ^10^, ^11^, ^12^, ^13^ have been carried out to compare the nonunion of femoral shaft fractures treated with AP and EN. However, there is no consensus as to which treatment should be the first choice. In this meta‐analysis, the nonunion of femoral shaft fractures treated with AP and EN was analyzed to better evaluate the clinical efficacy of the two methods in the treatment of femoral nonunion.

There has a little systematic evaluation of the efficacy of AP and EN in the treatment of femoral shaft fracture. This meta‐analysis was conducted to assess the results of randomized controlled trials (RCT) to compare the efficacy of AP and EN.

## Materials and Methods

We designed and conducted this meta‐analysis following the guidelines proposed by the Cochrane Handbook for Systematic Reviews of Interventions (http://handbook-5-1.cochrane.org/) and it was reported in compliance with the Preferred Reporting Items for Systematic Reviews and Meta‐Analyses (PRISMA) statement guidelines. As this study is based on previously published studies, ethical evidence and patient consent were not provided.

### *Search Strategy*

We searched all relevant RCT studies comparing the efficacy between AP and EN from PubMed, Embase, and the Cochrane Library from inception to April 2019. The MESH terms and keywords used in combination and separately in the search were as follows: nonunion, femoral fracture, femur, shaft, exchange, plate, and intramedullary nail. Only English‐written literature was included in the study. In addition, a manual search for references from review articles was performed to supplement the electronic database search.

### *Inclusion and Exclusion Criteria*

The inclusion criteria for the study were: (i) the design of the study was a randomized controlled study in humans; (ii) the subjects of study must be adults with femoral shaft fracture nonunion after IMN; (iii) interventions have to include both AP and EN for the treatment of nonunion of femoral shaft fractures; and (iv) the study should have sufficient follow‐up time. The exclusion criteria were as follows: (i) biomechanical experiments, case reports, review articles, or interventions did not accord with the inclusion criteria; (ii) studies of fractures in animals; and (iii) pathological fractures or infectious nonunions. Two investigators (XHC and SZH) independently screened the titles and abstracts of all articles.

### *Data Extraction*

The following data were extracted from each eligible study by the same two investigators (XHC and SZH), including name, number of participants, study design, interventions, and follow‐up time. A third investigator (PXK) checked the accuracy of the information extracted.

### *Risk of Bias Assessment*

Two investigators independently assessed the methodological quality of all potential articles. The judgments of investigators of bias were “low risk”, “high risk”, or “unclear risk” based on the following items: random sequence generation, allocation concealment, double blinding of participants and personnel, blinding of outcome assessment, incomplete outcome data, selective reporting, or other bias. In cases of disagreement, a third investigator (PXK) was consulted to make a decision.

### *Statistical Analysis*

The two investigators checked the input data from the included studies to ensure accuracy. Statistical analyses were conducted using the RevMan 5.3 software. For dichotomous outcomes, odds ratios (*OR*) with 95% confidence intervals (*CI*) were assessed in this meta‐analysis. For continuous data, we calculated the means and the standardized mean difference (*SMD*) with 95% *CI*. The *I* ^2^ statistic was tested to evaluate the statistical heterogeneity. *I* ^2^ > 50% was considered to have moderate heterogeneity^14^. A fixed‐effects model was applied when the *I* ^2^ statistic was >50%. In contrast, a random‐effects model was considered. *P* < 0.05 was considered statistically significant.

## Results

### *Study Characteristics*

All included RCT^9^, ^10^, ^11^, ^12^, ^13^ were published between 2010 and 2019. Five trials enrolled patients with nonunion of femoral shaft fractures and the sample size ranges from 18 to 190. Specifically, 276 samples were included in the control group and 232 samples were included in intervention‐group. In the intervention group, AP was used for treatment of nonunion of femoral shaft fractures, while in the control group, EN was used. Five RCT evaluate the outcomes through different assessment methods, such as: union, union time, intraoperative blood loss, complication rate, operation time, and mean postoperative draining volume. For union time and complications, all the articles are used in the methodology of evaluation, although the five articles have their own evaluation methods. In addition, four articles mentioned the basic parameters: study design, age, gender, assessment methods, and follow‐up time. Only one article did not provide the details about age and gender. The duration of follow‐up ranges from 3 to 217.2 months. The main characteristics are summarized in Table 1.

**Table 1:** The characteristics of included studies

Study	Year	Patients (n)		Age (years)		Gender (F/M)		Study Design	Assessment methods	Follow‐up (months)
		EN	AP	EN	AP	EN	AP			
Park *et al*.^10^	2010	7	11	42 (22–57)	43 (22–68)	6/1	8/3	RCT	Union rate, union time, operation times, complications	38 (26–84)/36 (25–82)
Kim *et al*.^9^	2010	20	4	NR		NR		RCT	Clinical union periods, complications	4.95 (3–9)
Ru *et al*.^11^	2015	87	93	46.5 ± 11.2	48.2 ± 8.4	48/39	52/41	RCT	Bone union, union time, blood loss, complication rate, operation time, mean postoperative draining volume	49.2 (12–85.2)
Ru *et al*.^12^	2016	92	98	46.6 (19–56)	48.8 (22–54)	51/42	53/45	RCT	Union, union time, the intraoperative blood loss, complication rate, operation time, mean postoperative draining volume	55.2 (12–217.2)
Lai *et al*.^13^	2019	70	26	35.79 ± 14.51	31.77 ± 11.97	36/34	18/8	RCT	Union rate, union time, operating time, intraoperative blood loss, complications	13.70 (3–50)/11.89 (4–32)

Five trials were selected and included in this meta‐analysis. Except for one article that did not provide details of age and gender, the four trials, respectively, provided the characteristics of the research subjects: patients, age, gender, assessment methods, and follow‐up time. Union rate, union time, operation time, blood loss, and complications were embedded into the study for assessment. AP, augmentative plating; EN, exchanging nailing. NR, no report; RCT, randomized controlled trial.

### *Characteristics of Included Studies*

**Figure 1:**
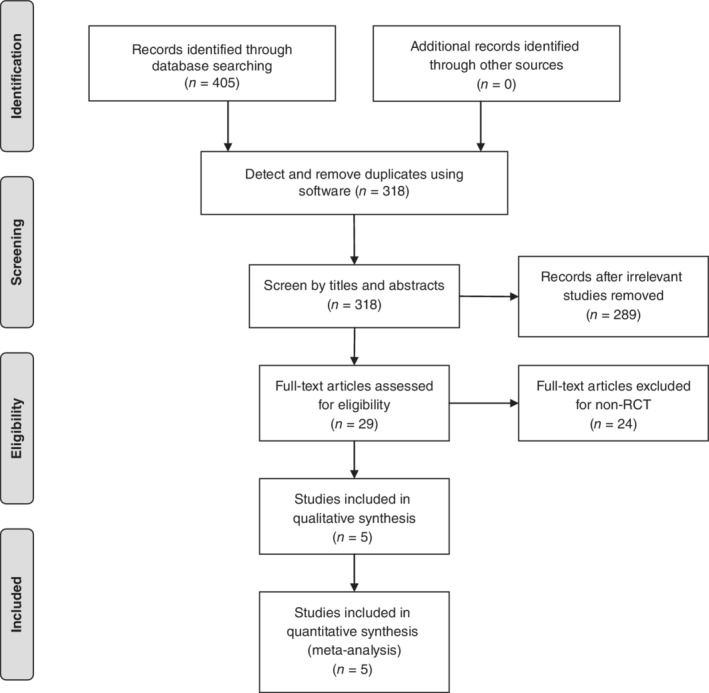
A flow chart of article selection for inclusion. A total of 405 studies were included in this meta‐analysis through a database search, and 376 articles were excluded by screening the abstracts and titles for duplicates, biomechanical experiments, case reports, reviews articles, and non‐comparative studies. Then, a total of 29 full‐text articles were assessed for eligibility. In the end, five RCTs with 508 patients were included in this meta‐analysis.

A total of 405 relevant articles were identified as potential inclusion studies through searching databases, and 376 articles were excluded by screening the abstracts and titles for duplicates, biomechanical experiments, case reports, reviews articles, and non‐comparative studies. Then, a total of 29 full‐text articles were assessed for eligibility. Specifically, 18 studies were excluded as they were not RCT. Six studies were excluded due to the uninteresting outcomes. Eventually, five RCT with 508 patients (276 from EN group, 232 from AP group) were included in this meta‐analysis. Details of the process for including the articles is shown in Fig. 1.

### *Risk of Bias in Included Studies*

**Figure 2:**
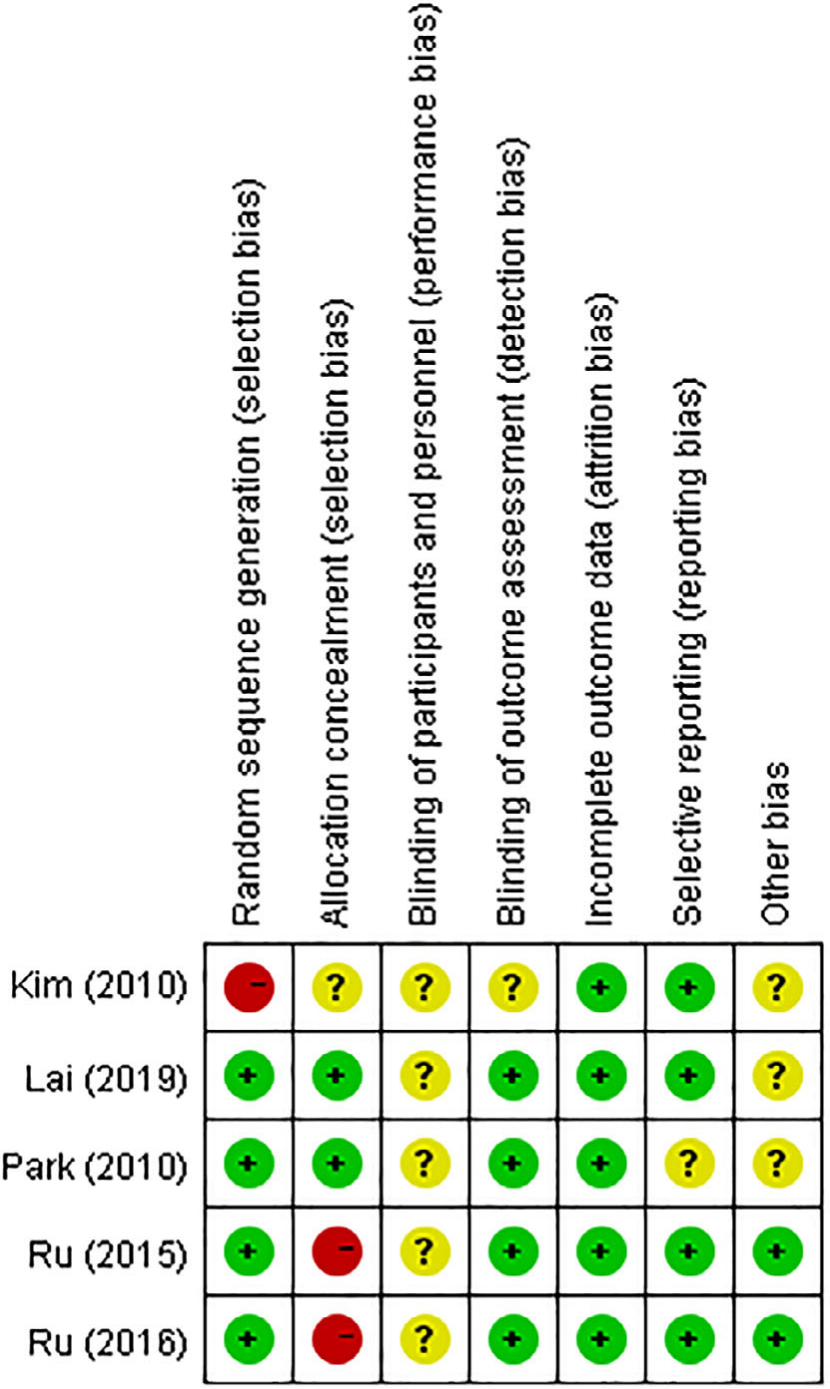
Risk of bias summary for each included study. The risk bias for included studies were assessed using the Cochrane Handbook. The red ball means “high risk,” the yellow ball means “unclear risk,” and the green ball means “low risk.”

**Figure 3:**
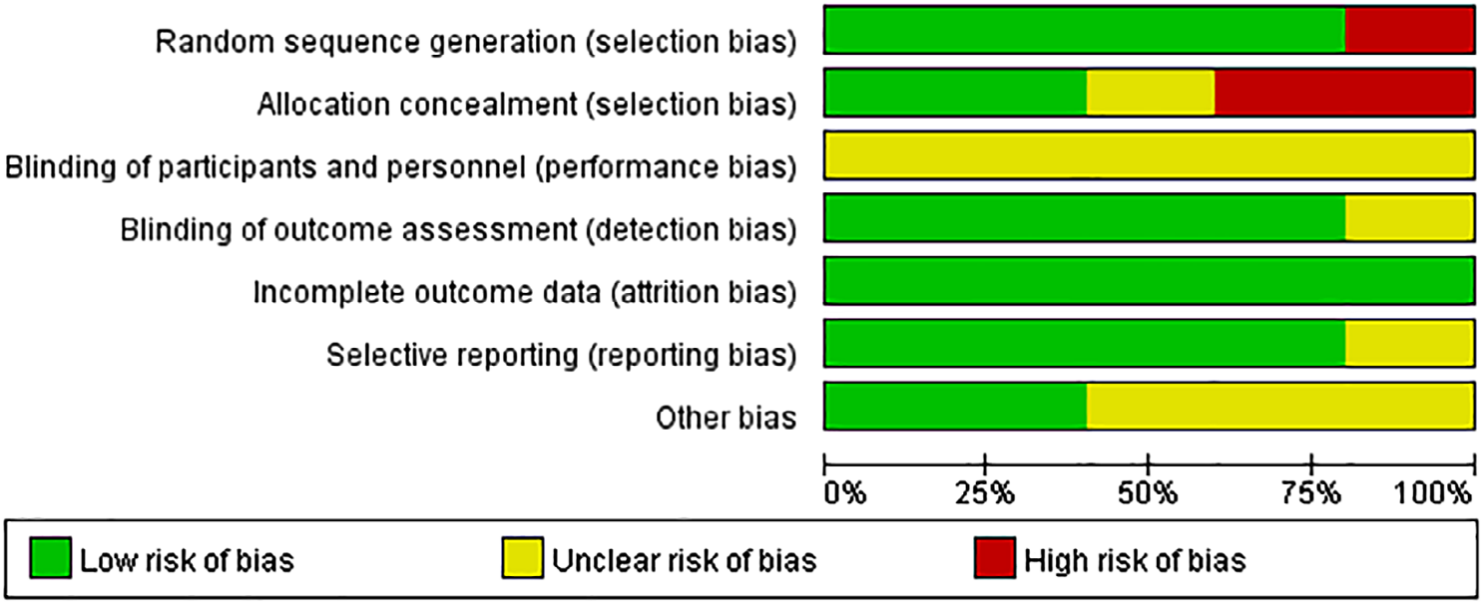
Risk of bias graph: review authors' judgments about each risk of bias item presented as percentages across all included studies. One trial did not provide details of random sequence generation, even though all trials in this study are randomized trial designs. One trial did not describe the method of concealing group allocation and two trials were assessed as “high risk.” Information of blinding of participants and personnel was not provided for five studies. Blinding of outcome assessment was unclear in one trial and selective reporting was unclear in another trial.

Five trials^9^, ^10^, ^11^, ^12^, ^13^ were assessed using the Cochrane Handbook, and the risk of bias of included studies is shown in Fig. 2 and summarized in Fig. 3. One trial^9^ did not provide details of random sequence generation, even though all trials in this study are randomized trial designs. One trial^9^ did not describe the method of concealing group allocation and two trials^11^, ^12^ were assessed as “high risk.” Information on blinding of participants and personnel was not provided for the five studies^9^, ^10^, ^11^, ^12^, ^13^. Blinding of outcome assessment was unclear in one trial^9^ and selective reporting was unclear in another trial^10^.

### *Outcome of Meta‐analysis*

The major adverse events, including union rate, union time, operation time, blood loss, and complications, were embedded into the study for evaluation.

#### *Union*

**Figure 4:**
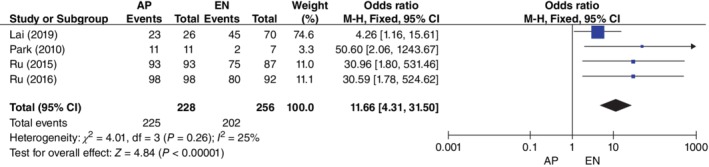
Forest plot of union rate between the augmentative plating (AP) group and the exchange nailing (EN) group. Four studies with 484 patients (228 from AP group and 256 from EN group) provided data on union rate. Data were pooled using a fixed‐effects model (M‐H Fixed). There was low heterogeneity among these studies (*I* ^2^ = 25%). *P* < 0.05, and the difference was statistically significant.

Four studies^10^, ^11^, ^12^, ^13^ with 484 patients (228 from the AP group and 256 from the EN group) provided data on union rate. The rate of femoral fracture union was 98.7% (225/228) in the AP group and 78.9% (202/256) in the EN group. There was low heterogeneity among these studies (*P* = 0.26, *I* ^2^ = 25%). Data were pooled using a fixed‐effects model and this result showed that the AP group had a significantly higher union rate than the EN group (*OR*, 11.66; 95% *CI*, 4.31–31.50; *P* < 0.01) (Fig. 4).

#### *Union Time*

**Figure 5:**
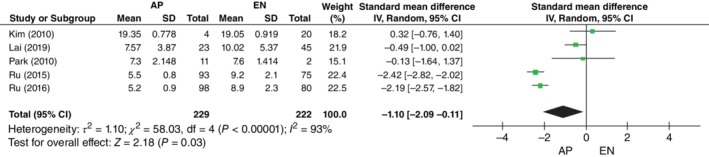
Forest plot of union time between the augmentative plating (AP) group and the exchange nailing (EN) group. Five studies with a total of 451 patients reported data on union time. Data were pooled using random‐effects analysis. *I* ^2^ = 93% represents high heterogeneity. Mean ± standard deviation (Std.) and 95% *CI* are shown. *P* < 0.05, difference was statistically significant.

All the studies^9^, ^10^, ^11^, ^12^, ^13^, with a total of 451 patients, reported data on union time in the AP group (229 patients) compared with the EN group (222 patients). The mean union time was 9.0 months in the AP group and 10.9 months in the EN group. Kim *et al*.^9^ recorded the union time in weeks, whereas the other four studies recorded the union time in months, so the comparison could only be done once the time was converted to months. Random‐effects analysis, with an *I* ^2^ of 93%, indicated that union time was shorter in the AP group (*SMD*, −1.10; 95% *CI*, −2.09 to −0.11; *P* = 0.03) (Fig. 5).

#### *Operation Time*

**Figure 6:**
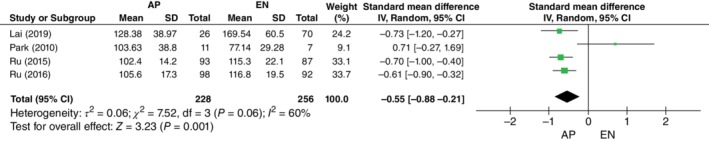
Forest plot of operative time between the augmentative plating (AP) group and the exchange nailing (EN) group. The operation time obtained in four studies with 484 patients was analyzed in the meta‐analysis. Data were pooled using random‐effects analysis. *I* ^2^ = 60% represents high heterogeneity. Mean ± standard deviation (Std.) and 95% *CI* are shown. *P* < 0.05, difference was statistically significant.

The operation time obtained in four studies^10^, ^11^, ^12^, ^13^, with 484 patients (228 in the AP group and 256 in the EN group), was analyzed in the meta‐analysis, and random‐effects analysis was adopted due to the high heterogeneity of the study (*I* ^2^ = 60%). The AP group showed a significantly shorter operation time when compared to the EN group (*SMD*, −0.55; 95% *CI*, −0.88 to −0.21; *P* < 0.01) (Fig. 6).

#### *Blood Loss*

**Figure 7:**
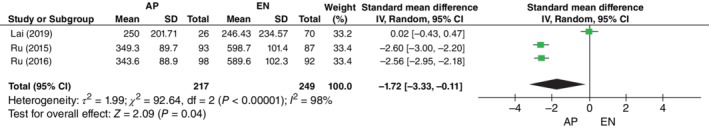
Forest plot of blood loss between the augmentative plating (AP) group and the exchange nailing (EN) group. The blood loss volume was reported in 3 studies with 466 patients. Data were pooled using random‐effects analysis. *I* ^2^ = 98% represents high heterogeneity. Mean ± standard deviation (Std.) and 95% *CI* are shown. *P* < 0.05, difference was statistically significant.

Blood loss volume was reported in three studies^11^, ^12^, ^13^, with 466 patients (217 in the AP group and 249 in the EN group). In view of the obvious heterogeneity in these results (*I* ^2^ = 98%), we adopt the random‐effects model. The meta‐analysis indicated that the AP group had significantly less blood loss compared to the EN group (*SMD*, −1.72; 95% *CI*, −3.33 to −0.11; *P* = 0.04) (Fig. 7).

#### *Complications*

**Figure 8:**
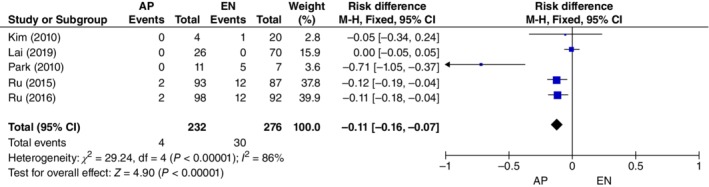
Forest plot of complications between the augmentative plating (AP) group and the exchange nailing (EN) group. Five studies of 508 patients (232 from the AP group and 276 from the EN group) reported the number of complications. Data were collected using a fixed‐effects model (M‐H Fixed). There was low heterogeneity among these studies (*I* ^2^ = 86%). *P* < 0.05, difference was statistically significant.

Five studies^9^, ^10^, ^11^, ^12^, ^13^ of 508 patients (232 from the AP group and 276 from the EN group) reported the number of complications (including infection and re‐nonunion). Overall, data collected using a fixed‐effects model (M‐H Fixed) (*I* ^2^ = 86%) and the study showed that the number of complications was significantly lower in the AP group than in the EN group (*OR*, −0.11; 95% *CI*, −0.16 to −0.07; *P* < 0.01) (Fig. 8).

## Discussion

The femoral shaft is one of the most common fracture types. The use of intramedullary nails effectively prevents rotation and displacement of fracture ends^15^. It should be noted that osteoporosis, improper treatment of open fractures, neglect of protection of hematopoietic function of broken ends, improper selection of fixation methods, and improper use of materials can easily lead to the occurrence of nonunion after fracture surgery.

Compared with other treatments, EN is considered a suitable choice for the treatment of nonunion after IMN for femoral shaft fractures^16^. However, conflicting reports on its success have been reported. Banaszkiewicz *et al*.^17^ indicated that significant complications occurred in 58% of patients after the EN, and further surgery was required in just under two‐thirds of the patients. In addition, AP is routinely used to treat femoral shaft nonunion with excellent results. Vaishya *et al*.^18^ found that all patients with femoral shaft nonunion healed without complications by retaining intramedullary nailing and AP. At present, the treatment of nonunion of femoral shaft fractures after IMN remains controversial, and there is no meta‐analysis and evaluation of AP and EN.

The present study was based on five RCT that includes 232 patients treated with AP and 276 patients treated with EN, and was designed to evaluate the relative advantages and clinical efficacy of AP and EN in the treatment of femoral shaft nonunion.

From this meta‐analysis, intraoperative blood loss and operative time were used to evaluate the methodological advantages during the surgery. The pooled analysis from three studies^11^, ^12^, ^13^ showed that there was significantly less blood loss during surgery in the AP group (*P* < 0.05). When further comparing operative times, the compiled data revealed a shorter time for the AP group than for the EN group (*P* < 0.01).

The greater blood loss and longer operative time in the EN group was due to the need to remove the original intramedullary nail before installing a new one. This is a complex operation compared to AP, which involves directly installing the plate. In addition, long‐term exposure to X‐rays can cause irreversible problems such as gene mutation and cancer. Excessive bleeding can also lead to decreased postoperative bone healing ability and increased healing time. Hemorrhage is the main cause of anemia after surgery, and massive hemorrhage can also cause hemorrhagic shock in patients.

Bundkirchen *et al*.^19^ concluded that hemorrhagic shock retards fracture healing during the early phase of the facture healing process in an *in vivo* mouse fracture model.

Besides, we extracted postoperative indications from this study, including union rate, union time, and incidence of complications. The pooled analysis from five studies^9^, ^10^, ^11^, ^12^, ^13^ indicated that the AP group had a significantly higher union rate than the EN group (*P* < 0.01). Lai *et al*.^13^ concluded that AP provided a significantly higher union rate than EN in treatment for femoral shaft aseptic nonunion. This is consistent with our conclusion. Moreover, union time was shorter in the AP group compared to the EN group (*P* < 0.05). Another important finding in this meta‐analysis was that a lower rate of complications for the AP group than for the EN group (*P* < 0.01). Complications in this study, including superficial infection and re‐nonunion, had a great negative influence on the fracture healing and increased hospital costs. Plate fixation was generally associated with low infection rates in some studies due to the gradual improvement of biotechnology^20^, ^21^. Of course, the choice of methods also needs to be determined according to the actual situation. Chen *et al*.^22^ suggested that retention of the intramedullary nail is performed if the fixation is stable and the infection is under control, but external fixation is most suitable for uncontrollable osteomyelitis or infected nonunion. In addition, nonunion is often accompanied by bone defects. Staged bone grafting is usually necessary when a bone defect is present^22^. As fracture healing is a natural repair process of the body, any interference factors will affect the process of fracture repair.

This is the first meta‐analysis looking at the most recent randomized controlled trials comparing the efficacy of AP and EN in nonunion of femoral shaft fractures after IMN. Of course, the present study cannot avoid the existence of restrictions, just like other meta‐analyses. First, the results were limited by the small number of patients (508) and a relatively short follow‐up time. Second, we could not conduct a subgroup evaluation to exclude this confounding issue due to the small variety of research. Furthermore, all of the included articles were in English, which may produce a language bias. Other potential problems were mainly manifested in the existence of invalid results data and publication bias. Despite these limitations, our quantitative assessment of the rates of union, blood loss, the time to operation and the incidence of complications provide a vital basis for surgical choices.

### *Conclusion*

In summary, our meta‐analysis demonstrates that both AP and EN methods have achieved good results in the treatment of nonunion of femoral shaft fractures after IMN. However, AP provides a shorter operative time and less blood loss during the surgery, and a higher union rate, a shorter union time, and a lower complication rate during the postoperative period (Table 2). Therefore, AP is found to be superior for nonunion of the femoral shaft after IMN. It is worth noting that bone grafting as a combined treatment for nonunion with bone defects. Given the limited number of patients enrolled in randomized managed trials, further carefully designed RCT, with larger pattern sizes, are vital to compare the efficacy of AP and EN.

**Table 2:** Comparison of outcomes between the AP and EN groups for nonunion of femoral shaft fracture

Groups	Union rate (%)	Union time (months)	Operation time (min)	Blood loss (mL)	Complications (%)
AP‐group	98.7	9.0	112.13 ± 23.49	314.3 ± 129.77	2.0
EN‐group	78.9	10.9	133.88 ± 34.03	478.24 ± 146.09	12.6
* *P* value	<0.05	<0.01	<0.01	<0.05	<0.01

* *P* < 0.05, difference was statistically significant.

The outcome assessment was compared between the AP group and the EN group in this meta‐analysis. AP, augmentative plating; EN, exchanging nailing.
